# Reply to Alizadeh’s Letter to the Editor Re: Lu, P.Y. et al., *Nutrients* 2017, *9*, 38

**DOI:** 10.3390/nu9070720

**Published:** 2017-07-07

**Authors:** Pei-Ying Lu, Long Shu, Shan-Shan Shen, Xu-Jiao Chen, Xiao-Yan Zhang

**Affiliations:** 1 Department of Geriatrics, Zhejiang Hospital, Xihu District, Hangzhou 310013, China; lupy007@sina.com (P.-Y.L.); shenshan305@163.com (S.-S.S.); 2 Department of Nutrition, Zhejiang Hospital, Xihu District, Hangzhou 310013, China; shulong19880920@126.com (L.S.); zxy19740804@sina.com (X.-Y.Z.)

To the Editor:

We have read the letter by Alizadeh regarding our article entitled “Dietary Patterns and Pancreatic Cancer Risk: A Meta-Analysis” as published in *Nutrients* in January 2017 [1]. The aim of this meta-analysis was to assess the associations between whole-diet and the risk of pancreatic cancer.

First, we are grateful to Alizadeh et al. for alerting us to the methodological limitations in this manuscript. We accept the methodological limitations in the present meta-analysis. Thus, in this reply, we have endeavored to respond to the point highlighted by Alizadeh. In our analyses, 32 studies (13 studies reported the associations between alcohol intake and pancreatic cancer) were finally included. As Dr Alizadeh says, there are only seven studies [2,3,4,5,6,7,8] reporting the associations between whole-diet and the risk of pancreatic cancer. However, we cannot ignore the effect of food groups on the risk of pancreatic cancer, although they do not represent whole-diet. To our knowledge, the association of diet with the risk of pancreatic cancer has been inconclusive. Besides, although some studies have reported the decreased risk of pancreatic cancer associated with fruits and vegetables, an international panel of experts concluded that the evidence for the association of fruit and vegetable consumption in relation to pancreatic cancer risk is limited and inconsistent. Thus, to minimize error, the authors ensured that the defined dietary patterns were similar with regard to the main factor loadings of foods, which are consumed within those whole-diets. For example, the first pattern, named as the healthy pattern, is characterized as having high loadings of foods such as vegetables, fruits, whole grains, olive oil, fish, soy, poultry and low-fat dairy. The second pattern, named as the “western-type” pattern, is characterized by a high consumption of, e.g., red and/or processed meat, refined grains, sweets, high-fat dairy products, butter, potatoes and high-fat gravy, and low intakes of fruits and vegetables. Higher meat consumption may reflect a western dietary behavior and lifestyle. Thus, we considered the meat intake in the studies of Taunk et al. [9], Nöthlings et al. [10], and Anderson et al. [11] as a western/unhealthy dietary pattern.

In conclusion, we show in this reply that the issue raised by Alizadeh et al was not ignored. But our findings are useful as an exploratory meta-analysis to confirm the significant associations between diet and pancreatic cancer and add to the existing literature supporting the concept that diet plays an important role in the prevention of pancreatic cancer.

We are grateful to Alizadeh for bringing to our attention to the methodological limitations in the conduct of the meta-analysis, and for giving us an opportunity to reassess our work from his perspective.
